# Supplementary material for: The rate of quasiparticle recombination probes the onset of coherence in cuprate superconductors
Source: Sci Rep. 2016 Apr 13;6:23610. doi: 10.1038/srep23610 (PMC4829850; doi:10.1038/srep23610)
Supplement: Supplementary Information [file srep23610-s1.pdf]

Supporting information for "The rate of quasiparticle recombination probes the onset of coherence in cuprate superconductors"

J. P. Hinton

Materials Science Division, Lawrence Berkeley National Laboratory, Berkeley, California 94720, USA
Department of Physics, University of California, Berkeley, California 94720, USA and
Department of Physics, University of California, San Diego, California 92093, USA

E. Thewalt, A. Lanzara, and J. Orenstein

Materials Science Division, Lawrence Berkeley National Laboratory, Berkeley, California 94720, USA and
Department of Physics, University of California, Berkeley, California 94720, USA

Z. Alpichshev, F. Mahmood, and N. Gedik

Department of Physics, Massachusetts Institute of Technology, Cambridge, MA 02139

J. D. Koralek and A. F. Kemper

Materials Science Division, Lawrence Berkeley National Laboratory, Berkeley, California 94720, USA

M. K. Chan, M.J. Veit, C.J. Dorow, L. Ji, and M. Greven

School of Physics and Astronomy, University of Minnesota, Minneapolis, Minnesota 55455, USA

N. Barišić

Institute of Solid State Physics, TU Wien, 1040 Vienna, Austria and
School of Physics and Astronomy, University of Minnesota, Minneapolis, Minnesota 55455, USA

D. A. Bonn, W. N. Hardy, and Ruixing Liang

Department of Physics and Astronomy, University of British Columbia, Vancouver, Canada. and
Canadian Inst. Adv. Res., Toronto, ON M5G 178, Canada

(Dated: January 10, 2016)

Fitting procedure

Here we describe details of the fits to $\Delta R(t)/R$ to demonstrate that the values of $\tau_{qp}(T)$ discussed in the main text are independent of the fitting procedure. Fig. S1 (a) shows time-resolved reflectivity data for five temperatures below, at, and above T_c for the Hg-1201 sample with $T_c =$ 71 K. The three-component fits, displayed in red, are of the form described in the text, $\Delta R = Ae^{-t/\tau_{qp}} - Be^{-t/\tau_r} + C$. Fig S1 (b) and (c) display the temperature dependence of the parameters extracted from these fits. For each temperature, the fit extends from the delay at which $\Delta R(t)$ first attains 50% of its peak value to $t = 8$ ps. The parameters B and τ_r account for both the finite rise time of ΔR and the presence of the negative PG component. These two contributions cannot be easily disentangled, but they can be captured in the fit by a single negative term in ΔR. As can be clearly seen, the structure in τ_{qp} near T_c is not present in the other fit parameters. This confirms that the peak in $\tau_{qp}(T)$ is representative of the rate of quasiparticle recombination and is not related to structure in the other parameters. The time-independent component C reflects the long-lived photo-induced heating of the system and the non-exponential decay that occurs at low temperatures. This component is much smaller than the exponential components and does not affect the other fit parameters.

In order to further demonstrate that the structure reported in τ_{qp} is not related to the parameters B and τ_r, we can limit the fit to an exponential decay plus constant, for example by considering ΔR only after it has decreased to 75% of its peak value. Fits to a two-component form $\Delta R = Ae^{-t/\tau_{qp}} + C$ are displayed in Fig. S2 (a) and the resulting fit parameters are compared to those from the three component fits in Fig. S2 (b)-(c). It is clear that the quantities of interest, A and τ_{qp}, are not affected by the details of the fit procedure.

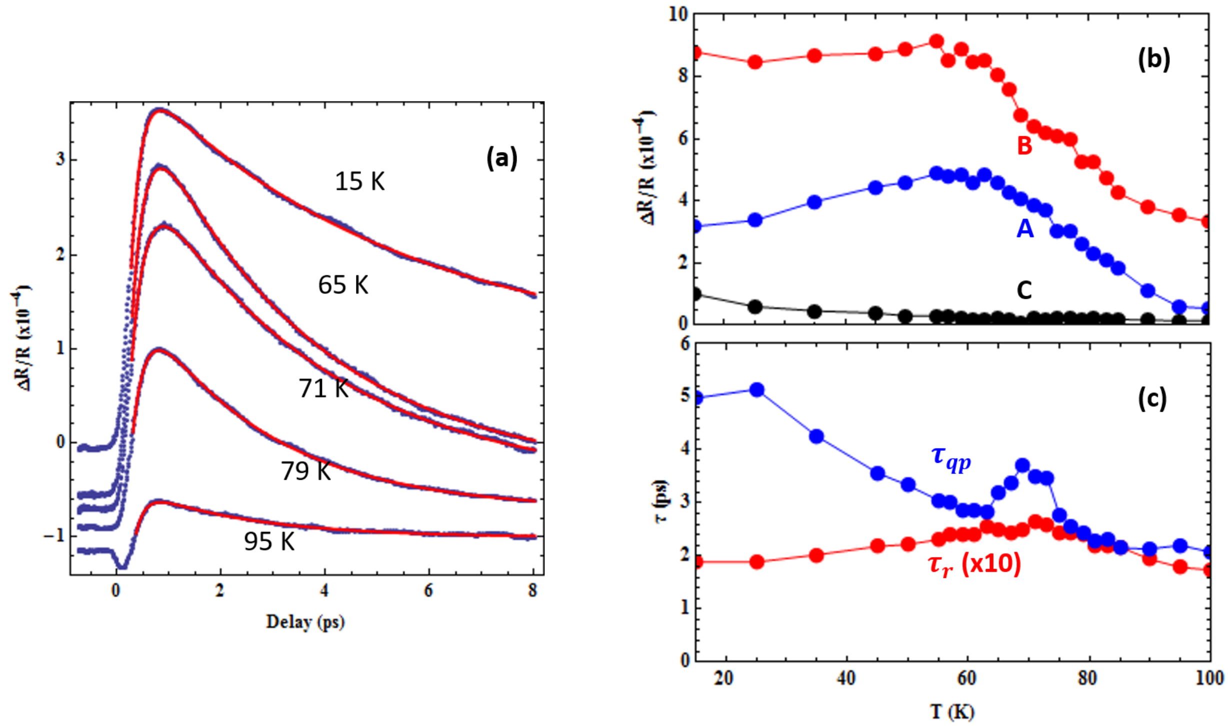

FIG. 1. (a) Select $\Delta R/R$ curves from the $T_c = 71$ K sample of Hg1201. The three-component fits are shown in red. (b) Temperature dependence of the three amplitudes A, B, and C. (c) Temperature dependence of the rise and decay time constants τ_r and τ_{QP}. τ_r is multiplied by 10 for easy comparison.

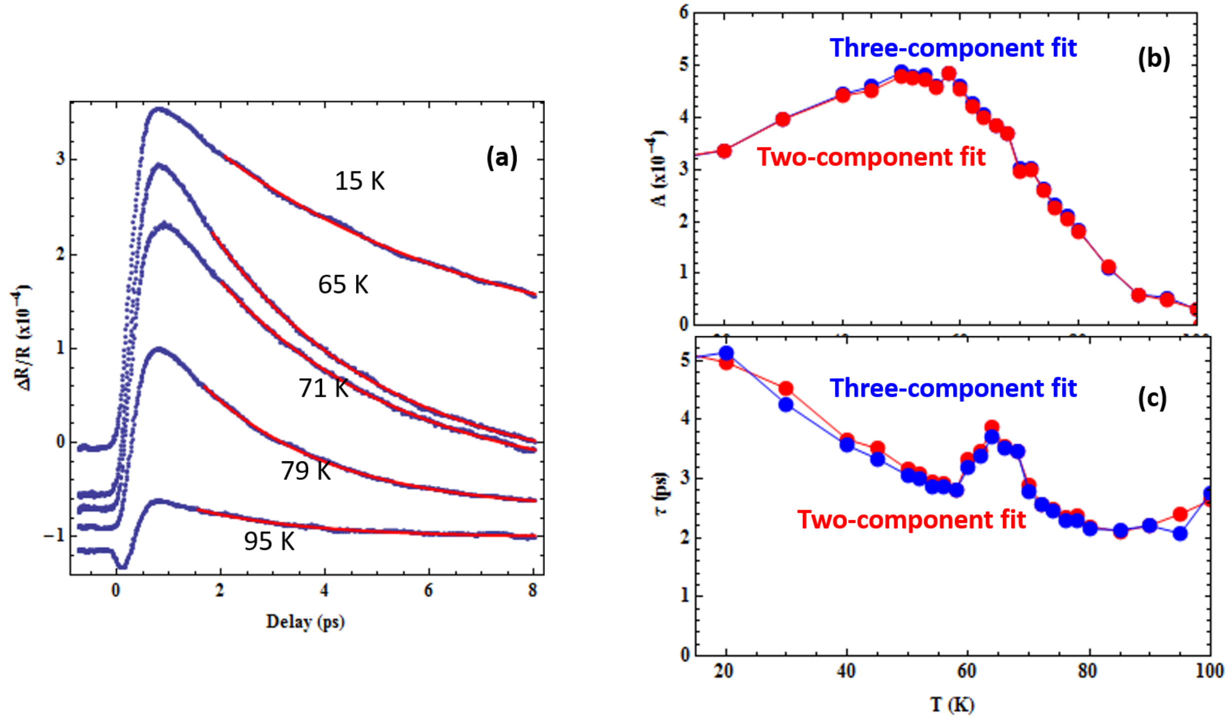

FIG. 2. (a) Select $\Delta R/R$ curves from the $T_c = 71$ K sample of Hg1201. The two-component fits are shown in red. (b) Comparison between $B(T)$ extracted from the three-component fit (blue) and the two-component fit (red). (c) Comparison between $\tau_{QP}(T)$ extracted from the three-component fit (blue) and the two-component fit (red).
